# Miscellany

**Published:** 1902-10

**Authors:** 


### Miscellany.

To produce Spring Temper in Swiss Broaches.—To draw Swiss broaches to a spring temper they should be placed on a steel, iron, or brass plate, one-eighth of an inch thick and about three inches square. This should be held by pliers or forceps over a spirit lamp, or Bunsen burner, and be kept continually moving over it, so as to keep the plate heated as uniformly as possible. The broaches should be watched very carefully, and when they become a pigeon-blue color they should be dropped into cold water.

---

An Oil-Stone Lubricant.—For oil-stones use one part glycerin and two parts alcohol. It keeps the surface clean and sharp-gritted. Oil thickens by use and exposure, and gums the stone.—H. W. Steele, *Items of Interest.*

---

Carved Solid Cusp for Gold Crowns.—At the clinic given at the Tri-State meeting held at Indianapolis Dr. J. E. Nyman demonstrated a very practical method of making solid cusps for shell crowns. It consisted of moulding the cusp in modelling compound to conform to the opposing tooth, then securing a mould in mouldine and casting a cusp of scrap gold. The results were good and artistic.—*Dental Review.*

---

Instructions for concentrating Dioxygen.—The Oakland Chemical Co. gives the following instructions for concentrating dioxygen:

"In a small, smooth porcelain dish or saucer pour about one ounce of dioxygen; then place it on a water-bath, or on a sandbath, or on the back of a stove where it will be subjected to a greater heat of not over 200° F., and cover the vessel with a piece of loose paper to protect the contents from dust. The solution slowly evaporates, and as the bulk decreases the strength increases. One ounce of a three per cent. solution evaporated to one-fourth of an ounce, which should be accomplished in from thirty to forty minutes, gives a remainder which will test about twelve per cent. This is generally strong enough for all purposes, but stronger solutions can be made by continuing the evaporation. This solution can be preserved for a considerable period in a loosely stoppered bottle, if not exposed to light.

"The advantage of this over other concentrated solutions of hydrogen dioxide is that the dentist never has to handle a dangerous product. When concentrated solutions are packed in tightly stoppered bottles, they frequently explode, and the accidents which have resulted from handling them are serious and numerous; with the above method, the solution is never in anything but an open or loosely stoppered bottle and no explosion is possible. The dentist can supply himself with small or large quantities on very short notice at less expense."

